# Oligodendrocyte death and myelin loss in the cuprizone model: an updated overview of the intrinsic and extrinsic causes of cuprizone demyelination

**DOI:** 10.1186/s13024-022-00538-8

**Published:** 2022-05-07

**Authors:** Martin Zirngibl, Peggy Assinck, Anastasia Sizov, Andrew V. Caprariello, Jason R. Plemel

**Affiliations:** 1 Faculty of Medicine & Dentistry, Neuroscience and Mental Health Institute, University of Alberta, Edmonton, Canada; 2 Wellcome Trust- MRC Cambridge Stem Cell Institute, University of Cambridge, Cambridge, UK; 3 Centre for Regenerative Medicine, Institute for Regeneration and Repair, University of Edinburgh, Edinburgh, UK; 4 Department of Clinical Neurosciences, Hotchkiss Brain Institute, University of Calgary, Cumming School of Medicine, Calgary, Canada; 5 Department of Medical Microbiology and Immunology, University of Alberta, Edmonton, Canada; 6 Department of Medicine, Division of Neurology, University of Alberta, Edmonton, Canada

**Keywords:** Cuprizone, Multiple Sclerosis, Demyelination, Oligodendrocytes, Inflammation, Cell death, Astrocytes, Microglia, CNS

## Abstract

The dietary consumption of cuprizone – a copper chelator – has long been known to induce demyelination of specific brain structures and is widely used as model of multiple sclerosis. Despite the extensive use of cuprizone, the mechanism by which it induces demyelination are still unknown. With this review we provide an updated understanding of this model, by showcasing two distinct yet overlapping modes of action for cuprizone-induced demyelination; 1) damage originating from within the oligodendrocyte, caused by mitochondrial dysfunction or reduced myelin protein synthesis. We term this mode of action ‘intrinsic cell damage’. And 2) damage to the oligodendrocyte exerted by inflammatory molecules, brain resident cells, such as oligodendrocytes, astrocytes, and microglia or peripheral immune cells – neutrophils or T-cells. We term this mode of action ‘extrinsic cellular damage’. Lastly, we summarize recent developments in research on different forms of cell death induced by cuprizone, which could add valuable insights into the mechanisms of cuprizone toxicity. With this review we hope to provide a modern understanding of cuprizone-induced demyelination to understand the causes behind the demyelination in MS.

## Background

Multiple Sclerosis (MS) is a chronic neurological disorder characterized by the loss of myelin or demyelination. Myelin is produced by oligodendrocytes in the central nervous system (CNS) wherein a single oligodendrocyte may myelinate up to 80 axon segments [1] to facilitate axonal signaling [1] and provide metabolic support to the axon [2]. Loss of oligodendrocyte and demyelination is associated with axonal damage [3–6]. Given the importance of myelin to CNS health, a wide body of MS research focuses on understanding the vulnerability of oligodendrocytes and their associated myelin sheaths to identify novel strategies to improve myelin regeneration, otherwise known as remyelination, and to slow neurodegeneration.

The disease course of MS is variable – different stages of the disease are characterized by distinct cellular mechanisms and inflammatory processes that depend on CNS-resident immune cells such as microglia, and the infiltration of peripheral immune cells, such as T- and B-cells. Primary-progressive MS (PPMS) is characterized by a linear worsening of symptoms from disease onset [7] and given that the blood–brain barrier (BBB) is mostly intact, CNS/cerebrospinal fluid -localized mechanisms likely facilitate demyelination [8, 9]. Relapsing–remitting MS (RRMS) is characterized by new lesions that cause distinct spikes in symptomatic disease that are followed by a recovery from disability [7, 10]. Relapsing stages of MS generally involve BBB breakdown and infiltration of T- and B-cells that then propagate inflammation to induce damage. RRMS often evolves into a progressive state with no symptomatic remission, termed secondary-progressive MS (SPMS) [7, 11]. Due to the different cellular mechanisms that characterize the different MS stages, modeling the disease as a whole is not feasible with one given model. Thus, three types of animal models are widely employed for modeling the different aspects of MS: (1) Virus-induced demyelination, (2) Experimental autoimmune encephalomyelitis (EAE), and (3) toxin provoked demyelination [12, 13]. Viral-induced demyelination is provoked by infection of mice with viruses such as Theiler’s murine encephalomyelitis virus (TMEV) or mouse hepatitis virus. This infection leads to inflammatory demyelination in brain and spinal cord, with a mixture of cluster of differentiation (CD)4^+^ and CD8^+^ T-cells, B-cells, microglia, and macrophages contributing to the demyelination [14].

To induce EAE, mice are injected with one or more myelin peptides in combination with an immune-boosting adjuvant and pertussis toxin, myelin reactive primed T-cells, or by expressing an autoimmune T-cell receptor [13]. These stimuli evoke an inflammatory autoimmune response against myelin and axons, facilitated by CNS infiltrating T-cells and monocytes that cause demyelination and disability [15, 16]. EAE is thought to model lesion formation and inflammatory injury characteristic of relapsing–remitting MS. However, the profound immune response and the stochastic appearance of demyelinated lesions make it challenging to decipher CNS intrinsic immune relevance to disease progression and remyelination (see [12, 13] for further reading). For these reasons, researchers may employ toxin-based animal models that limit the stochasticity of lesion formation and peripheral immune cell infiltration [17].

Toxin-based models of demyelination are primarily used to study mechanisms of primary demyelination and subsequent remyelination [18, 19]. Certain toxin-induced models of demyelination are initiated by the injection of toxic compounds such as lysophosphatidylcholine (LPC), lipopolysaccharide (LPS), or ethidium bromide into white matter areas to create a focal lesion at the injection site. An alternative toxin-based model of demyelination is the dietary consumption of cuprizone that causes demyelination of specific brain white matter regions, such as corpus callosum and hippocampus [20]. Among the toxin-based methods to induce demyelination, Bis-cyclohexanone-oxaldihydrazone, colloquially known as cuprizone, was found to be the most commonly used MS model in a recent systematic review and meta-analysis [21]. Cuprizone is a copper chelator that, when fed to rodents, results in a loss of oligodendrocytes and thus, myelin, in specific brain regions, with limited BBB disturbance and infiltration of peripheral immune cells. Despite cuprizone being first described in 1950 and the current widespread use of the toxin as a model to study demyelination and remyelination, little is known about how cuprizone elicits its toxic effects on the oligodendrocyte population.

To this end, there is a need to model demyelination and subsequent remyelination in animal models to enhance our understanding of oligodendrocyte susceptibility and to test potential pro-myelinating treatments that could be moved towards clinical therapies [22]. We argue that an improved understanding of the cuprizone model is vital for the use of this model in pre-clinical studies. To provide a better understanding, we showcase how early cuprizone experiments contributed to our understanding of how it damages oligodendrocytes and myelin. We discuss both cuprizone-induced oligodendrocyte-intrinsic mechanisms of demyelination and oligodendrocyte-extrinsic factors that can induce or exacerbate oligodendrocyte toxicity, with a focus on extrinsic immune-derived factors. We also summarize the diverse forms of oligodendrocyte cell death that occur in the cuprizone model as understanding oligodendrocyte death may illuminate mechanisms of cuprizone toxicity. We believe, those modes of action of cuprizone toxicity can be further categorized into three forms of pathology: (A) Oligodendrocyte cell death as a direct result of the effect of cuprizone on the oligodendrocyte population, which we call ‘primary oligodendrocytopathy’. (B) Oligodendrocyte cell death caused by activated astrocytes and microglia in response to oligodendrocyte damage, which we term ‘toxic innate immunity’ and (C) oligodendrocyte cell death caused by astrocytes and microglia following a direct effect of cuprizone on these cells, which we term ‘primary immunocytopathy’.

## Main text

### The cuprizone model – past and present

Considering how long and widespread cuprizone is used as a model for demyelination and remyelination [21], it is surprising that so little is understood regarding the mechanisms of its toxicity. The knowledge we do have about the mechanisms of how cuprizone elicits its effect is greatly influenced by findings obtained more than six decades ago. To understand how this still affects the way we think about cuprizone, we must explore how cuprizone was established as a model for MS.

#### The past – establishment of the cuprizone model of MS

Cuprizone was first described in 1950 by Gustav Nilsson, when he found that cuprizone is a sensitive indicator for copper, producing a colorimetric shift in the presence of copper [23]. In subsequent years, cuprizone was revealed to also produce severe systemic effects on the nervous system and peripheral organs in rodents. William Carlton first fed cuprizone to albino mice in the 1960s and described reduced growth of weanling mice, induced paresis, and terminated pregnancies following dietary consumption [24, 25]. These altercations coincided with brain edema, non-inflammatory demyelination, and astrogliosis in the cerebellum, cerebellar cortex, and medulla [24, 25]. In the late 1960s, others described mitochondrial abnormalities following cuprizone consumption in the mouse liver [26]. Cuprizone administration induces mitochondrial enlargement, severely alters metabolic rates, and impairs oxidative phosphorylation by reducing mitochondrial enzymes that contain copper as a cofactor, such as monoamine oxidase [27] and cytochrome c (Cyt c) oxidase [28, 29] in the brain of mice. Cuprizone treatment impaired hepatocytic mitochondrial function [30], which was thought to be a result of copper deficiency. Whether the consequences of cuprizone are directly related to copper chelation is unclear given that copper supplementation has limited capacity to reduce cuprizone toxicity to the CNS of mice [24].

Although most of the early experiments using cuprizone were conducted in mice, a number of studies demonstrated detrimental effects of cuprizone administration to the brain – similar to those in mice – of other rodents, such as rats [31–35], guinea pigs [31] or hamsters [36, 37]. Interestingly, in experiments using hamsters the cuprizone concentration needed to be increased to 3% or even 5%—compared to 0.2%—0.5% in experiments on mice or rat—to induce brain alterations. One very small study using non-human primates did not find brain demyelination in young cynomolgus macaques even after 18 weeks on a 3% cuprizone diet [38]. However, the lack of demyelination could be because even higher, but untested, doses of cuprizone are needed to induce demyelination in macaques. For this review, outside of these studies, unless stated otherwise, all other cuprizone research mentioned is carried out in mice.

The BBB of mice was found to be largely intact, as confirmed by various assays for leakage of components of the periphery into the CNS, including horseradish peroxidase [39, 40], immunohistochemistry [40], and radioactive tracers [41], which is why it was long believed that the cuprizone-induced demyelination occurs without peripheral involvement. Due to these early findings, metabolic disturbance of the mitochondrion was long thought to be the main reason for oligodendrocyte death and myelin loss. However, the early explorations of cuprizone toxicity used mice of varying strains, sex, and ages, which complicates interpretations of findings between experiments, thus highlighting the need for a more standardized approach.

#### The present – hallmarks of the modern cuprizone model

**Fig. 1:**
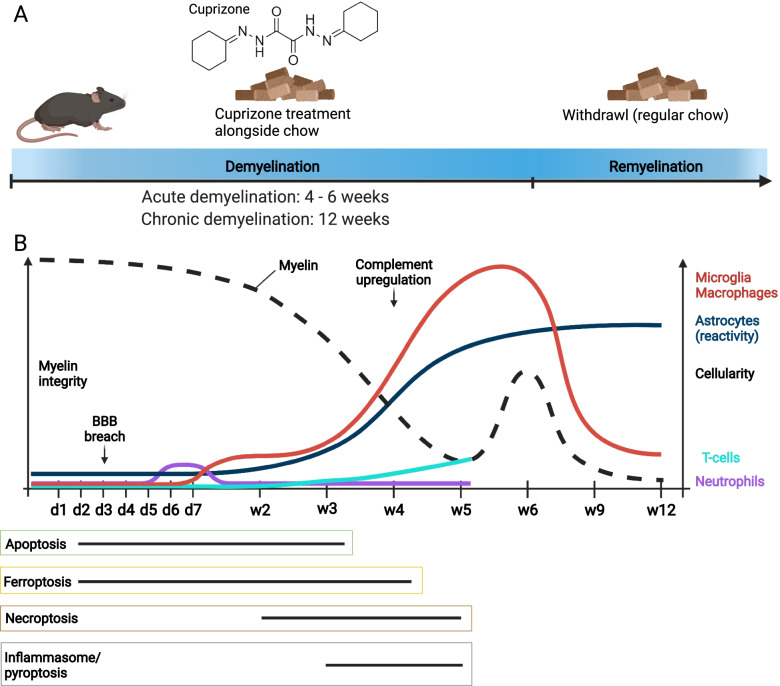
**A** Representation of commonly used cuprizone exposure protocol. Rodents are fed with concentrations of typically 0.2 – 0.3% (w/w) cuprizone in animal chow for up to 6 weeks to study acute demyelination and 12 weeks to study chronic demyelination. This is sometimes followed by a period of normal chow without cuprizone (withdrawl) to induce remyelination. **B** Time course of cuprizone intoxication in the corpus callosum. The dashed black line represents myelin integrity, and the colored lines represent cellularity (numbers of cells, unless stated otherwise) of microglia/macrophages in red, astrocytes in blue (here cellularity refers to reactivity), T-cells in teal, and neutrophils in purple. Below the timeline we show different modes of cell death observed in the cuprizone model, and their time of occurrence depicted by the black bar. Apoptosis (green box) occurs between day 2 and 3 weeks. Ferroptosis markers are elevated (yellow box) between day 2 and 4 weeks. Necroptosis (brown box) is seen in the cuprizone model between week 2 and week 5. Inflammasome-mediated cell death/pyroptosis (grey box) occurs between 3 and 5 weeks

To standardize the cuprizone model, Hiremath and colleagues introduced a protocol for lower-dose cuprizone administration (0.2% w/w) in C57BL/6 mice in 1998 [42, 43] that is still widely used today. Now, cuprizone is commonly administered for 4–6 weeks to study acute demyelination or up to 12 weeks to study chronic demyelination, and mice are occasionally followed after cuprizone withdrawal to study remyelination (Fig. 1A). Interestingly, endogenous repair processes occur despite the consumption of cuprizone [44]. For example, spontaneous remyelination, with up to 50% of myelinated axons and increased numbers of mature oligodendrocytes, can be seen after 6 weeks, despite continued exposure to cuprizone [45, 46]. The histopathological hallmark of the cuprizone model as standardized by Hiremath is oligodendrocyte death and subsequent demyelination in the corpus callosum, superior cerebellar peduncles, hippocampus [42, 46–54] and several other areas of the mouse brain (reviewed in [20]). In this model, myelin destabilization and loss begin at 2–3 weeks of cuprizone administration and peaks at week 4–5 [42], myelin levels stay low until at least week 12 [55] with continuous cuprizone feeding. This progressive demyelination is accompanied by robust astrogliosis and microgliosis (Fig. 1B). Astrogliosis begins coincident with demyelination after 2 weeks on cuprizone diet. Astrocyte densities peak around 5–6 weeks and stay high until 12 weeks. Microglia also proliferate and expand after 2 weeks, and their density peaks at 5—6 weeks [45, 55, 56]. Demyelination of brain white matter coincides with behavioral changes and loss of cognitive and motor function [57, 58]. While the cuprizone diet causes widespread demyelination, removal of cuprizone permits remyelination of the corpus callosum and cerebellar peduncles after a seven-week cuprizone diet [59] or even a longer seven-month diet, although remyelination is less efficient with chronic cuprizone consumption [60]. Despite this remyelination, persistent behavioral changes such as poor adaptive motor learning and motor function are apparent 4–7 weeks after cuprizone withdrawal, which is several weeks longer than is required for remyelination [57, 61]. Others found ongoing degeneration of the corpus callosum tracts continues despite robust remyelination [62, 63] suggesting ongoing dysfunction despite myelin repair.

Together, the adoption of a standardized cuprizone administration protocol improved inter-study reliability and by using the C57BL/6 inbred mouse strain, provided better options for conducting studies on transgenic mice, many of which are bred to a C57BL/6 background. By combining a standardized cuprizone model with transgenic mice, researchers are able to dissect genes, pathways and cell-specific roles in cuprizone toxicity more intimately. 

### Cuprizone-induced pathology mimics aspects of MS 

Histopathological analysis of post-mortem MS tissue revealed profound lesion heterogeneity both within and between MS patients [64–66]. Lucchinetti and colleagues categorized patterns of pathology into distinct lesion types, all of which contain T-cell, plasma cell, and macrophage infiltrates [65, 67]. Lesion types 1 and 2 are thought to be the product of T-cell-mediated autoimmune processes, which are commonly replicated with the EAE model. Lesion types 3 in contrast are displaying features of primary oligodendrogliapathy and heightened oligodendrocyte cell death, comparable to cuprizone-induced demyelination [65, 68]. Other features common to both cuprizone-induced and type 3 MS lesions are mitochondrial stress, selective loss of myelin-associated glycoprotein (MAG), lesions with indistinct borders that are not perivascular, pronounced glial activation, and lymphocyte infiltration [43, 46, 65, 69–71]. Type 3 lesions are also often found in the corpus callosum of MS patients, a brain region primarily affected by cuprizone [65].

Like in MS lesions, T-cells are found in cuprizone lesions. Indeed, cuprizone lesions and progressive MS lesions contain comparable, if relatively sparse, T-cell densities [70]. The source of the T-cells in progressive MS lesions is unclear given that the BBB remains largely intact despite the enriched lymphocytic trafficking [72, 73]. These lymphocytes may be remainders from lesions formed earlier in the disease or they may migrate from prominent intrathecal inflammation – a characteristic of progressive MS [74]. With cuprizone exposure, the recruitment of T-cells to the lesion site [70] may result from the decreased BBB integrity induced by cuprizone [75, 76]

Cuprizone toxicity produces profound demyelination in the white and grey matter of the brain that mirrors sites of MS pathology [46]. Corpus callosum demyelination is detectable by histology and magnetic resonance imaging (MRI) in MS patients [65]. Similarly, the medial aspect of the splenium of the corpus callosum and lateral aspect of its body are highly reliable sites of injury during intoxication with 0.2% cuprizone, prone to profound microglia/macrophage accumulation [54]. Thus, the corpus callosum has become a popular region for the study of white matter demyelination. Grey matter demyelination also presents in MS – cortical, and hippocampal atrophy is most severe in the progressive stages of MS and is a prominent source of permanent disability [77, 78]. Cuprizone toxicity is one of the few models of MS that produces grey matter demyelination, specifically in the cortex, hippocampus, and deep grey matter nuclei [79, 80]. Cortical grey matter demyelination is evident after 4 weeks of cuprizone treatment, but microglia/macrophage density in this region remains near baseline levels [54, 81, 82]**.** Likewise, cuprizone administration results in layer-specific cortical degeneration, with demyelination enriched within layers five and six [54]. Given that cuprizone administration induces both white and grey matter demyelination, it remains a highly relevant and useful model for studying the biological underpinnings of MS lesions.

Despite these similarities, there are some distinct differences between MS and the cuprizone model. For example, grey matter remyelination in MS is more efficient than white matter remyelination [83, 84]. However, upon cuprizone withdrawal, the white matter corpus callosum is remyelinated nearly to control levels within a week. In contrast, the grey matter of the cortex does not efficiently remyelinate in the same period, suggesting that grey matter remyelination may be slower [85]. The slower cortical remyelination may relate to technical challenges in measuring structures with vastly different myelination prior to cuprizone treatment, but this remains to be confirmed. Transgenic models that fate-map new myelin will be essential to better understanding grey matter demyelination and remyelination. Another difference between type 3 and cuprizone lesions is that MS lesions lack complement protein deposition [65], which is a feature of cuprizone toxicity [85]. Despite the striking similarities between cuprizone and MS lesions, an incomplete understanding of the mechanisms of cuprizone limits its full potential as a preclinical model for identifying next-generation therapeutics for MS.

### Death from within: how oligodendrocyte intrinsic mechanisms could lead to death during cuprizone treatment

Although MS is generally described as an autoimmune and inflammatory disease of the CNS, some argue that MS may initially reflect a non-inflammatory degenerative disorder that affects myelin and in doing so, begins a cascade that elicits the more commonly reported inflammatory response (for review see [8]). In line with this, researchers explore whether cuprizone acts directly on oligodendrocytes/the myelin sheath. Cuprizone is a copper chelator and considering that dysregulation of copper homeostasis in humans can lead to neurodegenerative diseases such as Menkes disease [86, 87], Wilson’s diseases [88] and is thought to contribute to Alzheimer’s disease and Parkinson’s disease [88, 89], better understanding potential mechanisms underlying demyelination mediated by copper chelation is necessary to better understand cuprizone toxicity.

Copper levels in the brain of cuprizone-treated mice are altered [28, 90, 91], though the extent of this change is unclear, as studies on copper levels in the brains of cuprizone-treated mice are contradictory. Using spectrophotometry Gilles Venturini reported a roughly 40% decrease of copper (Cu^2+^) in the brain of Swiss mice treated with cuprizone for 3–4 weeks [28], while others reported an increase in copper after 1 week (Cu^2+^ and Cu^+^) [90] and 3, 6 and, 9 months (total free and bound) [91] following cuprizone treatment. Similar to the inconclusive findings regarding the copper levels after cuprizone treatment, the actual chemistry of the copper-cuprizone complex is disputed as well. Some studies suggest hydrolysis of cuprizone in aqueous solutions to ‘monohydrazone cuprizone’ and multiple possible copper-cuprizone complexes in solution [23]. For example, Gustav Nilsson’s discovery that cuprizone changes color in copper solutions suggests that it can bind Cu^2+^ [23]. In later experiments cuprizone was shown to precipitate as a Cu^2+^-cuprizone complex when in high concentrations of copper. This Cu^2+^-cuprizone complex could not cross the intestinal epithelial barrier in an ex vivo experiment on mice intestines, which was thought to cause systemic copper deficiency [92]. Messori and colleagues proposed that two monohydrazone cuprizone molecules form a complex with the toxic Cu^3+^ [93]. A study on the redox properties of copper-cuprizone complexes confirmed the Cu^3+^-cuprizone complex and furthermore demonstrated that cuprizone can stabilize the toxic Cu^3+^ [94], which may be a source of oxidative stress.

The unresolved questions surrounding the effect of cuprizone treatment on brain copper levels and complex chemistry make it difficult to judge if the copper chelating property of cuprizone plays a role in cuprizone-induced demyelination. Indeed, there are a few studies suggesting a chelation independent mechanisms of cuprizone. In an in vitro investigation of the cuprizone inhibition of pig-plasma benzylamine oxidase—containing copper and pyridoxal phosphate as cofactor [95]—Lindström and Pettersson found that cuprizone interacts but does not extract copper from the enzymes active site [96]. Cuprizone likely covalently binds the other cofactor pyridoxal phosphate, which prevents enzymatic activity [96]. Taraboletti and colleagues confirmed this cuprizone-pyridoxal phosphate reaction and demonstrated cuprizone inhibits an aminotransferase by this same mechanism [97].

Systematically investigating the copper-cuprizone complex chemistry and copper chelation independent mechanisms may help unravel the mechanisms of cuprizone toxicity. Early after cuprizone initiation and in the context of minimal inflammation, changes in myelin composition and/or lipid polarity have been detected well in advance of oligodendrocyte cell body damage giving weight to the idea that cuprizone may have direct, albeit subtle, effects on myelin [79, 98, 99] – the human relevance of such altered myelin biochemistry prior to overt demyelination has since been confirmed [98]. In fact, the first signs of oligodendrocyte death are seen 2 days after initiation of cuprizone treatment [100, 101], which is several weeks earlier than the accumulation of other cell types at the site of injury [42]. In the following sections, we will review several theories on how cuprizone treatment leads to oligodendrocyte death from within, here defined as intrinsic death.

#### Mitochondrial dysfunction in oligodendrocytes as cause for oligodendrocyte death

Oligodendrocytes are highly specialized cells that produce myelin sheaths to ensure proper axonal signal transmission [102]. To maintain the myelin sheaths, oligodendrocytes require a high energy supply to synthesize sufficient amounts of the necessary lipids and proteins [103]. Oligodendrocytes also store large quantities of iron within ferritin [104, 105], but have low levels of the important reactive oxygen species (ROS) reducing agent glutathione (GSH) [106], which suggests that oligodendrocytes may be especially vulnerable to changes in metabolic rates and ROS. Therefore, the disturbance of oligodendrocyte mitochondria, the resultant energy shortage, ROS accumulation, and disturbance of lipid and protein synthesis, is discussed as a mode of action of cuprizone toxicity [20, 107, 108].

**Fig. 2:**
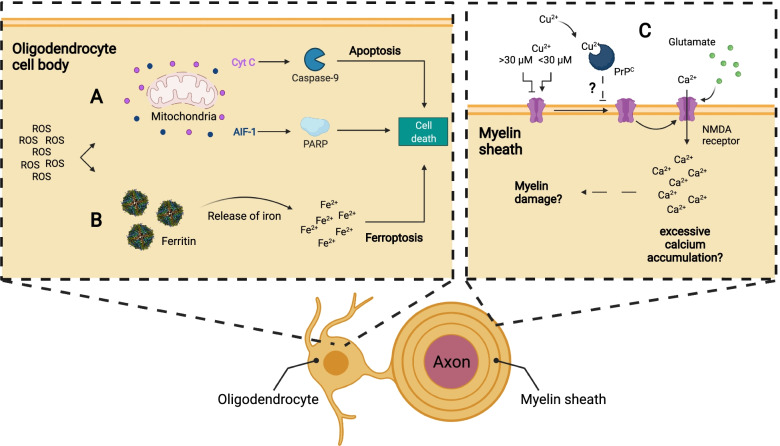
Various oligodendrocyte intrinsic mechanisms could lead to cell death of the oligodendrocyte during cuprizone intoxication. **A** Elevated levels of ROS can lead to permeabilization of mitochondria and subsequent release of cytochrome C and apoptosis-inducing factor-1. Cytosolic cyt c can activate caspase-9 and thus induce apoptosis in the oligodendrocyte, while apoptosis-inducing factor leads to cell death through PARP. **B** ROS reduces iron stored in ferritin from Fe^3+^ to Fe^2+^, which leads to iron release from ferritin. Free Fe^2+^ and ROS can induce lipid peroxidation, a hallmark of ferroptotic cell death. **C** Glutamate may reach toxic levels and act via NMDA receptors to permit excessive Ca^2^ influx. NMDA receptor activity can be modulated by copper. Depending on the concentration of copper present, NMDA receptor channel function is enhanced or inhibited. Also, PrP^C^ can inhibit the NMDA receptor in the presence of copper. Elevated levels of glutamate and changes to copper concentration in the brain as a result of cuprizone administration therefore might increase Ca^2+^ accumulation in the cell to a toxic level and myelin degradation/oligodendrocyte death

Cuprizone ingestion alters mitochondria in the CNS of mice [27–29, 109]. In vitro, cuprizone lowers the mitochondrial transmembrane potential of oligodendrocytes, but does not affect microglia, astrocytes, and neurons [110] – neither does cuprizone kill rat oligodendrocytes in vitro [111]. In vivo, 3 weeks of cuprizone administration results in morphological disturbances in oligodendrocyte mitochondria in the form of megamitochondria [109]. Enlarged megamitochondria are thought to reflect a compensatory response to elevated levels of ROS, based on evidence in vitro*.* Treatment of rat hepatocytes with hydrogen peroxide or ROS-inducing chemicals triggers a megamitochondria phenotype [112], which is reversed by buffering with free radical scavengers [113]. Megamitochondria can buffer a certain concentration of ROS, but if levels are kept high for too long, megamitochondria become permeable, swell, and release cyt c and apoptosis-inducing factor-1 (AIF-1) into the cytosol [114–116]. Cyt c released from mitochondria binds Apoptotic protease activating factor-1 (APAF-1) and subsequently activates caspase-9 [117] to trigger apoptosis [118–120] (Fig. 2A). After 3 weeks on a cuprizone diet, AIF-1—a regulator of Poly (ADP-ribose) polymerase (PARP) mediated cell death [121]—translocates into the nucleus of oligodendrocytes of treated mice, which is a step necessary for apoptosis. Inhibition of PARP prevents oligodendrocyte death and demyelination of the corpus callosum [71]. After 5 weeks of cuprizone diet, mitochondria in the corpus callosum of treated mice swell and release mitochondrial cyt c [122]. It is difficult to know the exact timeline of mitochondria swelling, cyt c release, and AIF-1 nuclear translocation and how this regulates early oligodendrocyte death as these early timepoints have not yet been investigated (Fig. 2A).

ROS also directly induce oligodendrocyte death (e.g., ferroptosis [101]). Staining the corpus callosum with 8-OhdG reveals that oligodendrocytes contain oxidized nucleotides – an indication of elevated ROS [123] – after 1 week of cuprizone treatment [124]. Oligodendrocytes are particularly sensitive to ROS given their heightened storage of Fe^3+^ within ferritin. The ROS superoxide reduces Fe^3+^ to Fe^2+^ to liberate the iron from ferritin in vitro [125]. This free iron can react via the Fenton reaction to produce hydroxyl radicals to generate toxic 4-Hydroxynonenal (4-HNE) and malondialdehyde (MDA), which are products of lipid peroxidation. Lipid peroxidation is responsible for ferroptotic cell death (Fig. 2B) and may be another mode of oligodendrocyte death (described in more detail below).

In addition to elevated levels of ROS, cuprizone consumption alters mitochondrial enzymes in a way suggestive of metabolic dysfunction. For example, cuprizone administration reduces the mitochondrial enzymes monoamine oxidase (as early as 3 days [27]) and cyt c oxidase [28, 29] in the brain of mice, both of which require copper for catalysis [126]. A recent in vivo study used MRI to describe significantly reduced ATP levels in the brain after 1 week of cuprizone treatment [124], further suggesting metabolic decline. Lowered mitochondrial enzymes could account for the decreased metabolic rates of mitochondria during cuprizone intoxication.

Despite clear mitochondrial and metabolic changes within oligodendrocytes following cuprizone consumption, how these alterations relate to oligodendrocyte death remains unclear. For example, impaired mitochondrial metabolism alone does not induce oligodendrocyte death in vivo [127]. Oligodendrocyte-specific deletion of *Cox10,* which encodes a crucial component for cyt c oxidase assembly and is necessary for oxidative phosphorylation, failed to cause demyelination or neurodegeneration even at 14-months of age. These COX10-deficient oligodendrocytes maintained axonal thickness without a change in the number of oligodendrocyte progenitor cells (OPC) or astrocytes and did not induce microglial activation, suggesting that oligodendrocytes can function without oxidative phosphorylation. Oligodendrocytes with impaired oxidative phosphorylation instead boost aerobic glycolysis, as indicated by increased lactate levels [127]. Lactate is likely shuttled to the underlying axons as a source of trophic support [128] or used to support myelination [129]. The possibility that aerobic glycolysis can sustain oligodendrocytes without reduced oxidative phosphorylation suggests that other stressors compound mitochondrial function to initiate oligodendrocyte death. Confusingly enough, cuprizone also does not kill oligodendrocytes in vitro [111], suggesting that cuprizone alone might not be enough to induce oligodendrocyte cell death in vivo.

#### Myelinic glutamate excitotoxicity

Glutamate is the main excitatory neurotransmitter in the brain [130]. Postsynaptic glutamate acts on N-Methyl-D-aspartic acid receptors (NMDAR), which opens a non-selective cation channel on neurons to permit calcium or sodium ion influx [131]. Calcium-dependent downstream signaling of NMDAR is important for modulation of synaptic plasticity and neuronal survival [132]. Despite the importance of glutamate and NMDAR for the healthy brain, overactivation of glutamate receptors leads to toxic levels of intracellular calcium, which can result in cell death [133, 134], for which the term excitotoxicity was coined [135].

In MS, glutamate is elevated prior to the formation of lesions and is correlated to lesion formation [136]. Similar to MS lesions, cuprizone consumption leads to elevated levels of total glutamate in the hippocampus (~ 10% increase based on in vivo ^1^H magnetic resonance spectroscopy) and corpus callosum (~ 60.8% increase based on glutamate-weighted chemical exchange saturation transfer) of rats after 7 weeks of cuprizone treatment [137, 138], which may result in excitotoxicity. The pathways that elicit elevated glutamate levels remain unclear. It may be that the unitary strength of glutamatergic synapses increases which in turn release more glutamate into synapses. One of the factors influencing synaptic strength is the level of vesicular glutamate transporter (VgluT) 1 [139], which loads synaptic vesicles with glutamate [140, 141]. Cuprizone treatment increases VgluT1 levels after 5 weeks [142] and may lead to increased release of glutamate from the synapse [143]. In contrast, both the NMDAR subunit NR2A and glutamate aspartate transporter (GLAST) – two factors that counteract excitotoxicity – are upregulated in the corpus callosum of cuprizone-fed mice after 5 weeks [144]. NR2A upregulation was correlated to neuroprotection in an in vitro study on NMDA-mediated excitotoxicity in primary rat retinal cells [145]. GLAST is upregulated in astrocytes following cuprizone consumption and likely mediates astrocytic clearance of glutamate from the synaptic cleft [144, 146, 147]. Elevation of GLAST in astrocytes is likely a protective response to increased glutamate levels [148, 149]. Taken together, we know now that glutamate is elevated, but so too are molecules that counteract glutamate excitotoxicity.

Aside from glutamate concentration, the activity of its receptor NMDAR also mediates excitotoxicity. Ions such as magnesium [150], zinc [151], and copper [152] regulate NMDAR activity. For example, copper potentiates the NMDAR current at concentrations < 30 µM while inhibiting it above 30 µM in rat cerebellum granule cells in vitro [152]. Proteins, such as the cellular prion protein (PrP^C^) also regulate NMDAR activity [153, 154]. PrP^C^ binds copper with high affinity and inhibits NMDAR in the presence of copper [155, 156] to prevent glutamate-mediated excitotoxicity. PrP^C^ is present in myelin and oligodendrocytes [157, 158] and thus may prevent excitotoxicity in the cuprizone model, though there is no direct evidence. In summary, cuprizone-mediated changes in brain copper levels can both increase and decrease NMDAR activity to promote or inhibit excitotoxicity respectively. As mentioned, measurements of brain copper levels following cuprizone treatment were rather inconclusive [28, 90, 91] and therefore it is unclear if copper concentrations decrease sufficiently to promote excitotoxicity via NMDAR. Further, copper levels are often expressed as µg_copper_/g_dryweightbrain_, which makes it difficult to compare changes in copper levels following cuprizone treatment between in vitro and in vivo models. Even though there is evidence that cuprizone could lead to excitotoxicity in oligodendrocytes and neurons, the precise impacts of cuprizone on NMDAR-mediated cell death are not fully understood but could provide a mechanism by which glutamate in the lesion can induce myelin damage (Fig. 2C).

#### Amino acid starvation results in reduced synthesis of myelin components

**Fig. 3:**
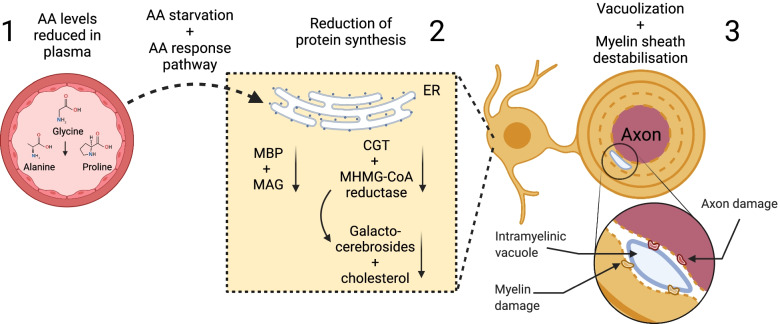
Cuprizone induced myelin loss could be due to reduced myelin protein production and the subsequent destabilization and vacuolation of the myelin sheath. (1) Cuprizone administration leads to reduced levels of certain amino acids (Glycine, Alanine, and Proline) in the blood plasma. This amino acid starvation could lead directly or indirectly – through the amino acid response pathway – to reduced protein synthesis in the oligodendrocyte. (2) In the cuprizone model several proteins are reduced, that are either part of the myelin sheath or enzymes that produce components of the myelin sheath. For example, MAG and MBP are reduced – both are crucial stabilizers of the myelin sheath. CGT and HMG-CoA reductase are reduced as well. CGT is crucial in producing Galactocerebrosides. HMG-CoA is involved in the cholesterol synthesis pathway. Both Galactocerebrosides and cholesterol are important parts of the myelin sheath membrane. Lack of MAG, MBP, galactocerebrosides, and cholesterol could lead to myelin sheath destabilization, formation of intramyelinic vacuoles, and subsequence myelin and axonal damage degradation

Cuprizone consumption may alternatively induce demyelination by reducing myelin protein and lipid synthesis which destabilizes the myelin sheath. For example, Morell and colleagues found that the messenger ribonucleic acid (mRNA) of various myelin proteins, such as MAG and myelin basic protein (MBP), decreases in the first week after cuprizone diet in rats by ~ 75% and ~ 50% respectively [159, 160]. Reduced production of myelin protein mRNA may be due to overall reduction of transcription and protein synthesis as a result of peripheral amino acid starvation. After only 4 days of cuprizone consumption, plasma levels of alanine, glycine, and proline decline. This amino acid starvation may trigger the amino acid response pathway, which decreases overall mRNA expression and protein synthesis [161]. Taraboletti and colleagues also found reduction of several amino acids and related metabolites in the corpus callosum of cuprizone treated animals after 2 weeks [97], which might be a cause for reduced protein synthesis. Ceramide galactosyltransferase (CGT) and β-Hydroxy β-methylglutaryl-CoA (HMG-CoA) reductase are two enzymes that are reduced following cuprizone consumption [159, 160]. CGT is a key enzyme in galactocerebroside synthesis and HMG-CoA reductase in cholesterol synthesis. Galactocerebrosides are the most abundant glycolipid of myelin [162] and cholesterol is an important part of the myelin sheath [163]. Thus, both are important for myelin sheath stability. The reduced production of crucial myelin proteins and proteins involved in myelin lipid synthesis following cuprizone treatment could lead to a lack of those components in the myelin sheath and subsequent destabilization of the myelin sheath. Sheath destabilization can result in vacuole formation within the myelin sheath and the periaxonal space, which could lead to oligodendrocyte and axonal degeneration [164, 165]. However, given that mRNA levels of myelin proteins are reduced at 1 week, when oligodendrocyte death is ongoing, the lower synthesis may be a consequence rather than a driver of oligodendrocyte toxicity (Fig. 3).

### Death from the outside: How oligodendrocyte extrinsic mechanisms could lead to death during cuprizone treatment 

Cuprizone treatment also evokes accumulation of various cells of the innate and adaptive immune response, which is accompanied by expression of pro-inflammatory cytokines and other inflammatory modulators. Those cells and molecules create a destructive environment, that can further aggravate or potentially initiate the demyelination process. In the following sections, we will showcase multiple cell types and inflammatory molecules that are known to contribute to oligodendrocyte death and demyelination in the cuprizone model.

#### The contribution of CNS resident cells to inflammatory demyelination in the cuprizone model

Understanding the CNS resident mechanisms of demyelination is of interest to MS research because not all demyelination in MS is linked to infiltration of peripheral immune cells [8]. Cuprizone treatment elicits a largely CNS-restricted immune response following injury to oligodendrocytes and myelin breakdown, making it an ideal model to study CNS intrinsic immune responses following demyelination.

##### Oligodendrocyte lineage cells are inflammatory cells

**Fig. 4:**
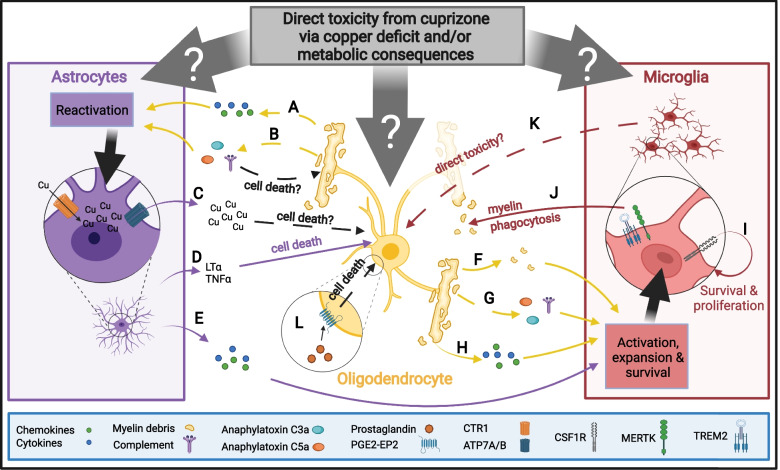
Proinflammatory response of CNS intrinsic cells resulting from cuprizone intoxication. Cuprizone might directly affect oligodendrocytes, astrocytes, and microglia. Cuprizone-induced demyelination immune response involves multiple – in part overlapping – steps. **A** As oligodendrocytes die, they release various cytokines and chemokines may lead to activation of astrocytes. **B** Complement is potentially produced or activated by damaged oligodendrocytes or from myelin breakdown. Activated complement produces anaphylatoxin C3a and C5a, which in turn can attract and activate astrocytes and microglia. Complement may also be directly cytotoxic to oligodendrocytes. **C** Reactive astrocytes may contribute to copper storage and redistribution in the CNS. The high affinity copper transporter CTR1 mediated copper influx into the astrocytes, while ATP7A/B are mediating copper efflux. This may create a locally toxic copper accumulation or release of factors that exacerbate oligodendrocyte death. Reactive astrocytes also produce (**D**) cytotoxic chemokines such as LTα and TNFα or (**E**) proinflammatory cytokines that lead to expansion and activation of microglia. Microglia are also activated when oligodendrocytes release (**F**) myelin debris, (**G**) by complement and anaphylatoxin, and (**H**) cytokines released from the injured oligodendrocyte. **I** Activated microglia require CSF1R stimulation for survival and proliferation. **J** Microglia are important for phagocytosis of myelin debris, for which TREM2 and MERTK are crucial. **K** Activated microglia may exhibit direct toxicity towards oligodendrocytes, (**L**) Other inflammatory mediators that could lead to oligodendrocyte death such as PG E2 via the PGE2-EP2 receptor

Historically, oligodendrocyte lineage cells that include oligodendrocytes and OPCs were regarded as the victims of inflammatory brain damage. However, oligodendrocyte lineage cells are now recognized to produce immunomodulatory factors that alter CNS inflammation. Oligodendrocyte lineage cells produce many different chemokines, cytokines, antigen presentation complexes, and complement proteins both in vitro and in vivo (reviewed in [166]). In MS lesions and EAE mice, oligodendrocyte lineage cells express immune-modulatory genes such as the major histocompatibility complex (MHC)-II, a protein crucial for antigen presentation [167]. Both oligodendrocytes and OPCs express MHC-II in response to Interferon (INF) and present antigens to regulate the proliferation and cytokine production of CD4^+^ T-cells [167]. Like EAE, cuprizone may induce immune-modulating properties in oligodendrocytes. The treatment of cultured oligodendrocytes with sodium azide—an inhibitor of the mitochondrial respiration—models altered mitochondrial function during cuprizone treatment and leads to increased mRNA levels of several cytokines and chemokines. These factors include potent inflammatory regulators such as Interleukin (IL)-6, Leukemia inhibitory factor (LIF), C-X-C motif chemokine ligand (CXCL)1, C–C motif chemokine ligand (CCL)5, and Colony stimulating factor (CSF) 1 [168]. Notably, CXCL1, CCL5, LIF, and CSF1 regulate microglia activation, survival, and migration to the site of injury [169–172] (Fig. 4H). Oligodendrocytes also produce IL-6 in vivo, a roughly fivefold increase in gene expression compared to control, after 2 days of cuprizone treatment [168]. Despite its pro-inflammatory properties, IL-6 protects against mitochondrial damage following bacterial infection [173] and is necessary for the repair of alcohol-induced mitochondrial deoxyribonucleic acid (DNA) damage [174]. The cytokine IL-6 seems to be neuroprotective in MS [175] and over expression of IL-6 in astrocytes preserves oligodendrocytes and reduces the severity of demyelination and numbers of microglia following cuprizone treatment [176, 177]. While oligodendrocytes are now recognized as mediators of CNS inflammation, it remains challenging to understand the consequences of their involvement given that the released factors may have both protective and degenerative functions.

##### Astrocytes are critical for cuprizone-induced demyelination

Astrocytes become reactive following oligodendrocyte damage and release cytokines, chemokines, and other factors that contribute to cuprizone-induced demyelination (Fig. 4). Astrocyte reactivity occurs throughout cuprizone treatment with morphological changes beginning as early as day 5 of cuprizone treatment. Astrocytes become hypertrophic and hyperplastic between weeks 1 and 2 of cuprizone administration, before myelin loss. Astrocyte reactivity is associated with increased activity of oxidative enzymes (glutamate dehydrogenase, lactate dehydrogenase, NAD(P)H dehydrogenase, NADH dehydrogenase) [42, 178, 179] which may contribute to ROS mediated toxicity. By week 3 of cuprizone administration, there is a roughly twofold increase in astrocyte numbers [42, 54] (Fig. 1), which is likely a critical modulator of inflammation given that the number of reactive astrocytes is concomitant with the severity of demyelination [42, 48].

Strategies that limit astrocyte reactivity reduce demyelination for cuprizone-treated animals. For example, knockout mice for receptor tropomyosin receptor kinase B (TrkB) show reduced astrocyte reactivity and in turn, demyelination [180]. TrkB is enriched within reactive astrocytes and stimulating astrocytes with a TrkB ligand in culture promotes the nuclear localization of the prototypical proinflammatory signaling pathway molecule Nuclear factor kappa B (NF-κB). The canonical NF-κB signaling pathway is a critical component of the proinflammatory astrocytic response induced by cuprizone [181]. The canonical NF-κB pathway may be activated by a TrkB ligand [180] but also by other cytokines, such as Tumor necrosis factor (TNF) [182]. In mice deficient in inhibitor kappa B kinase 2 (IKK2) – IKK2 is a subunit of IκB which lies downstream of NF-κB activity [182] – cuprizone treatment elicits less severe astrocyte and microglia reactivity and induces less myelin loss. IKK2 knockout mice also express lower levels of pro-inflammatory cytokines, including IL-1β, TNFα, CCL2, CCL3, and CXCL10, after 5 weeks of cuprizone treatment. Removing IKK2 from non-microglial CNS cells also prevents the loss of oligodendrocytes at weeks 3 and 5 compared to wild-type animals treated with cuprizone, suggesting that NF-κB pathway-derived cytokines aggravate demyelination in the cuprizone model [181]. Similarly, astrocyte-specific inhibition of NF-κB by overexpressing IκBα – the dominant-negative inhibitor of NF-κB – under the glial fibrillary acidic protein (GFAP)-promoter, prevents myelin loss and reduces TNFα, IL-1β, and CCL2 [181]. In summary, the NF-κB pathway mediates proinflammatory responses of astrocytes following cuprizone consumption and may be essential for an astrocytic role in demyelination.

The ablation of astrocytes during cuprizone treatment significantly decreases astrocyte reactivity and microgliosis, while sparing myelin, increasing oligodendrocytes density and improving motor function. Astrocyte ablation during cuprizone treatment also reduces the expression of CXCL10 in mice [183] – an important attractant for microglia during cuprizone treatment [184–186]. Astrocytes were also shown to secrete CXCL10 in vitro following stimulation with IFN-γ or TNF-α [185]. This suggests that astrocytes may promote microglial accumulation during cuprizone-induced demyelination. However, CXCL10 is also expressed by oligodendrocytes after 1 week of cuprizone treatment, suggesting that oligodendrocytes themselves may also help attract microglia to the area of demyelination [186]. Loss of CXCL10 does not alter astrocyte reactivity, but instead, reduces demyelination, microglial reactivity, and axonal damage after three weeks of cuprizone diet [186], thus linking this astrocyte-derived chemokine to microglia recruitment and demyelination (Fig. 4E).

Astrocytes are attracted to the site of injury and aggravate demyelination through the production of proinflammatory cytokines and chemokines. Certain astrocyte-released cytokines may induce oligodendrocyte cell death, such as TNFα, while others like CXCL10 can attract microglia to the site of injury. Astrocytes also produce Lymphotoxin α (Ltα) during cuprizone treatment [56], which is directly cytotoxic to oligodendrocytes in vitro [187]. Ltα is elevated in astrocytes, but not microglia, during cuprizone treatment and its astrocytic loss delays cuprizone-induced demyelination, oligodendrocyte loss, and microglia infiltration, suggesting that Ltα is an astrocytic cytokine that potentiates cuprizone toxicity (Fig. 4D).

Astrocytes also regulate copper levels in the brain by transporting and redistributing copper from endothelial cells into the CNS. CNS copper uptake is regulated via the high affinity copper uptake protein 1 (CTR1) whereas copper release from cells is regulated by two copper-transporting P-type ATPases (ATP7A and ATP7B) [188]. Astrocytic expression of CTR1, ATP7A, and ATP7B increases 1 week after cuprizone exposure [180]. Dysregulation of the copper transport system may be potentially toxic in the cuprizone model, as stimulating CTR1 expression in astrocytes along with high copper stimulus in vitro primes astrocytes such that once they return to basal conditions, they release factors—possibly copper—that are toxic to oligodendrocytes [180]. Whether copper or other factors are responsible for oligodendrocyte toxicity remains unclear, as do the consequences of these factors in vivo (Fig. 4C).

Together, astrocyte reactivity and accumulation at the site of injury precedes demyelination. As oligodendrocytes die, astrocytes produce cytokines such as TNFα and LTα that may kill oligodendrocytes. Astrocytes also produce chemokines that promote microglia expansion to the site of injury to further propagate the CNS intrinsic immune response (Fig. 4A-E).

##### Microglia are critical for cuprizone-induced demyelination

Microglia are highly responsive cells that respond to factors like myelin or oligodendrocyte debris following cuprizone-induced demyelination (Fig. 4F). Microglia also drive cuprizone-induced demyelination [189]. Microglia become reactive as early as 1 week of cuprizone treatment and sustain activity throughout demyelination [189–191]. In addition to expressing markers indicative of their reactivity, microglia also expand their numbers into the peak of demyelination observed after 3–5 weeks on a cuprizone diet [42], though, for unknown reasons, microglia numbers decline afterward despite continuing cuprizone diet [55, 56] (Fig. 1B).

One critical role for microglia during demyelination is the clearance of myelin debris, which acts as an inhibitor of remyelination [192–194]. Microglia accumulate in response to demyelination to consume myelin debris [195, 196]. Myelin debris is sufficient to induce the expansion and reactivity of microglia [197] (Fig. 4F). Myelin debris clearance during cuprizone consumption is regulated by receptor tyrosine kinases, such as MER proto-oncogene, tyrosine kinase (MERTK) that recognize phosphatidylserine on apoptotic cells [198, 199]. Loss of MERTK does not attenuate demyelination but instead impairs microglia recruitment and remyelination [198]. Similarly, triggering receptor expressed on myeloid cells (TREM) 2, a phospholipid sensing receptor, is expressed by microglia, sustains microglia activity, and mediates myelin debris consumption [200]. In mice lacking TREM2, fewer microglia accumulate in the corpus callosum during demyelination due to reduced proliferation which in turn reduces myelin debris clearance [200–202]. Mice lacking TREM2 also experience greater axonal damage after cuprizone treatment. Conversely, TREM2 agonists accelerate myelin clearance in the cuprizone model to facilitate remyelination [202] (Fig. 4J). Together, these findings suggest that TREM2 mediates neuroprotective functions during cuprizone-induced demyelination.

Despite the positive roles of microglia in response to cuprizone, a complete absence of microglia prevents cuprizone toxicity suggesting that these cells are also pathogenic during cuprizone-induced demyelination [189]. Microglia require CSF1 receptor (CSF1R) signaling for ongoing survival [171, 172] (Fig. 4I) and in the CNS, CSF1R is restricted to microglia [203]. The use of CSF1R antagonists ablates microglia, providing a tool to understand the role of microglia during disease [171]. PLX3397 is a CSFR1 inhibitor that ablates microglia with minimal upregulation of inflammatory genes [204], suggesting that inflammation is not overtly induced. Ablation of microglia with PLX3397 during cuprizone treatment reduces the loss of oligodendrocytes and the severity of demyelination in the corpus callosum after 3 weeks and 5 weeks [189, 205]. Pre-treatment of mice with PLX3397 for two weeks before cuprizone treatment to ablate microglia to low levels and continued PLX3397 treatment maintains microglia at very low numbers during cuprizone treatment. The pronounced and prolonged microglia ablation completely prevented cuprizone-induced demyelination with no substantial alterations to myelin ultrastructure [189]. However, it is still unknown how microglia drive cuprizone-mediated demyelination. Surprisingly, promoting the expansion of microglia by treating mice with CSF1 during cuprizone diet also reduced demyelination [172], demonstrating the complicated role of microglia during cuprizone intoxication. Taken together, microglia initiate demyelination potentially through direct toxicity or the expression and secretion of cytokines (Fig. 4K) but may also protect axons and promote remyelination during cuprizone consumption.

##### The complement system during cuprizone toxicity

In innate immune responses, the complement system acts as a first-line of defense against pathogens, but it also modulates other innate and adaptive immune cells [206]. Given its immunoregulatory functions, the complement system unsurprisingly is involved in cuprizone-induced demyelination. The complement complex C1q is comprised of C1qA, C1qB, and C1qC and facilitates the first step of classical complement pathway activation.[207]. The C1q complex is increased during the first 4 weeks of cuprizone consumption and accumulates in the corpus callosum during demyelination [208]. The gene expression of the component proteins of C1q and other complement proteins, namely C3a receptor and C4, are upregulated at 5 weeks of cuprizone [209]. Coincidentally, endogenous Complement receptor 1-related Gene/Protein y (Crry)—an important inhibitor of complement-mediated injury [210]—is reduced during demyelination [208], suggesting that complement may be disinhibited during cuprizone intoxication. The overexpression of soluble Crry (sCrry) in astrocytes is neuroprotective as it spares myelin in the corpus callosum and decreases microglia infiltration by week 4 after cuprizone exposure [208].

Anaphylatoxin C3a and C5a—downstream products of the complement pathway [211]—function as chemoattractants for neutrophils [212], macrophages [213], astrocytes [214], and microglia [215–217] (Fig. 4B&G). When overexpressed in astrocytes, these factors enhance demyelination and increase microglia numbers after cuprizone exposure. Indeed, treatment of a microglia cell line with C3a and/or C5a in vitro resulted in increased expression of the proinflammatory cytokines and chemokines CCL4, CCL5, CCL11, and IL-6 [218]. During cuprizone-induced demyelination, the complement proteins C3a and C5a increase the numbers of microglia present at the site of injury and might stimulate microglia to express pro-inflammatory cytokines [218]. There is also evidence that complement can be directly cytotoxic to oligodendrocytes in culture [219–221]. Given that anaphylatoxins regulate leukocyte phagocytosis [222], they may also alter microglia phagocytosis. Taken together, complement proteins indirectly aggravate demyelination by promoting the accumulation of proinflammatory microglia and astrocytes (Fig. 4B&G) at the lesion site and may directly induce oligodendrocyte death.

##### Cytokines and chemokines contribute to demyelination

Chemokines and cytokines are critical regulators of cuprizone-induced demyelination. Several inflammatory cytokines and chemokines such as CCL2, CCL3, CCL5, and CXCL10 are increased within the first week of cuprizone treatment, while others are increased later during demyelination such as CCL3, CCL4, C–C motif chemokine receptor (CCR) 5 [100, 186, 223]. Both CXCL10 and CCL2 peak early (~ week one) and decline after, while CCL3 expression increases with intoxication length [100, 186]. TNF expression levels are increased after one week of cuprizone-induced demyelination in vivo [224] and TNFα^−/−^ mice showed a delay in demyelination in the cuprizone model [56], consistent with a role for TNF to exacerbate cuprizone-induced demyelination. Given that TNF directly induces oligodendrocyte death in vitro [225, 226], it may directly participate in cuprizone-induced demyelination in vivo. However, TNF is also required for remyelination [224]. Many other immune factors are involved in cuprizone-induced demyelination. CCL3 knock-out mice have less microglia/macrophage accumulation and less demyelination compared to control mice on the cuprizone diet [223]. Also, loss of CCL10 largely prevented demyelination, oligodendrocyte death, microglia, and astrocyte activation and axonal damage at week 3 on the cuprizone diet [186]. Several other knockout studies showcase the influence of cytokines on demyelination, oligodendrocyte death, astrogliosis, and microgliosis in the cuprizone model, which are listed in Table 1 (modified table from [107]).

**Table 1:** List of cytokine and chemokine transgenic mouse lines used in the cuprizone model

Cytokine/Chemokine	Demyelination	Oligodendrocyte death	Microgliosis	Astrogliosis	Citation	
Conditional Overexpression (OE)						
CNS injected lentiviral expressed – Oncostatin M (OSM)	Less	Decreased	Decreased	Decreased	[227]	
GFAP expressed- C3a	More		More	More	[218]	
GFAP expressed – C5a	More		More	More	[218]	
GFAP expressed – IL-6	less severe after 5, reduced removal of degraded MBP	no difference	less severe	less severe	[176]	
GFAP expressed – IL-6	less severe 6 and 12 weeks in lateral cerebellar nuclei; CC not assessed	less severe at 12 weeks	induces specific activation state		[177]	
GFAP- expressed Platelet-derived growth factor (PDGF)-α	Normal				[228]	
GFAP expressed – sCrry	Less		Less		[208]	
GFAP expressed – IL-17A	more severe at 3 weeks		more severe		[229]	
GFAP conditionally expressed – IFN-γ	Normal	Normal			[230]	
GFAP conditionally expressed – IFN-γ PKR-like ER kinase (PERK) + /–	Normal				[231]	
MBP expressed -I FN-γ	Less	Less	Less	Less	[232]	
Conditional knockout (cKO)						
CX3CR1 cKO – CSF1R			reduced at 5 weeks			[233]
Constitutive knockout						
CCL2/3–/–	Less only in cortex, not CC	Less in cortex	Unaltered	Decreased in cortex	[234]	
CCL3–/–			Unaltered		[186]	
CCL3–/–	Delayed		Delayed	Delayed	[223]	
CCR2–/–	Unaltered	Unaltered			[235]	
CCR2–/–			Normal microgliosis but reduced macrophage infiltration		[236]	
CX3CR1–/– express GFP within locus			unchanged	Reduced expression of proinflammatory cytokines	[75]	
CX3CR1–/–	Decreased	Unaltered	Decreased		[235]	
CXCL10–/–	Decreased	Decreased	Decreased		[186]	
CXCR2–/–	Normal	Normal	Normal	Normal	[237]	
CXCR2–/–	Less	Less			[238]	
CXCR3–/–	no difference		less severe	less severe	[239]	
IFN-β–/–	Less severe	More	diminished		[240]	
IFN-a-Receptor–/–	Normal	Normal	Normal	Normal	[241]	
IFN-γ-Receptor–/–	Less	Normal	Delayed		[242]	
IL-17–/–	Less		Less		[243]	
IL-17-Receptor–/–	Less		Less		[243]	
IL-18–/–	Delayed	Delayed	Delayed	Delayed	[244]	
IL-1ß–/–	Normal	Normal	Normal	Normal	[244, 245]	
Interferon regulatory factor-8 (IRF-8)–/–	Less	Less	Delayed		[246]	
LIF–/–	More	More			[247]	
Ltα–/–	Delayed	Delayed	Delayed	Normal	[56]	
Ltß-Receptor–/–	Delayed		Delayed		[248]	
OSM-Receptor–/–	More	Increased			[227]	
p75 neurotrophin receptor (p75NTR)–/–	Normal	Normal			[249]	
TNFα–/–	Normal	Delayed	Normal		[224]	
TNFα-Receptor1–/–	Normal				[224]	
TNFα-Receptor2–/–	Normal				[224]	
TNF-like weak inducer of apoptosis (TWEAK)–/–	Delayed	Less	Delayed	Normal	[250]	
PDGF-α-Receptor + /–	Normal				[251]	

##### Prostaglandin involvement during demyelination

Prostaglandins (PG) are a family of lipids made at the sites of tissue damage that modulate inflammation [252]. PG are derived from arachidonic acid, an unsaturated fatty acid produced from phospholipids in the plasma membrane by phospholipase A2. Subsequently, arachidonic acid is converted to PG via Cyclooxygenase (COX)-1 and COX-2 [253]. During cuprizone consumption, COX-2 expression increases after 1 week and PG levels increase after 5 weeks in the cortex [254]. Indeed, COX-2^−/−^ mice and mice treated with celecoxib—a selective COX-2 inhibitor [255]—experience less oligodendrocyte death and demyelination in the corpus callosum on a cuprizone diet. At the same time, the PG E2 receptor 2 subtype (PGE_2_-EP2) localizes in apoptotic oligodendrocytes in the cortex, suggesting an involvement of PG in oligodendrocyte death in the cuprizone model. Inhibition of PGE_2_-EP2 with a selective antagonist also reduces cuprizone-induced demyelination in mice [191]. However, inhibition of PGE_2_-EP2 only reduces demyelination when started at the same time as cuprizone treatment, not when started during peak demyelination suggesting a role for PG during the initiation of demyelination (Fig. 4L).

The increased presence of PG in areas of cuprizone-induced demyelination underlines the importance of inflammation for cuprizone pathology. PG are also elevated in cerebrospinal fluid of people with MS [256, 257] and COX-2 is upregulated in demyelinating MS plaques [258, 259], suggesting a potential role of those inflammatory mediators in MS.

#### The contribution of peripheral immune cells to inflammatory demyelination

Apart from inflammatory cells from within the CNS, there is evidence that also peripheral immune cells/components might play a role in cuprizone-induced demyelination. Initially, it was thought that the BBB was intact during cuprizone-induced demyelination, which would limit infiltration of peripheral cells. In contrast, recent research using fluorescently tagged dextran (70 kDa) or Evans Blue tracer injection demonstrates BBB permeability as early as 3 days after cuprizone treatment [75, 76]. Gene expression analysis of corpus callosum tissue shows upregulation of *Tnf*, *Il-1b*, *Ccl2*, and *Il-6*, cytokines and chemokines involved in BBB breakdown, as early as 5–7 days of cuprizone treatment. Conversely, the expression of genes encoding the tight junction proteins that maintain the BBB, namely claudin-5, occludin, ZO1/tight junction protein-1, cadherin-1, and cadherin-5 decrease initially after 5 days and decreases further by 5 weeks on a cuprizone diet. Microglia likely promote BBB breakdown given that loss of the microglia C-X-C motif chemokine receptor (CXCR) 3 ameliorated both the CNS-wide rise in TNF, IL-1β, and CCL2 and the reduction in tight junction gene expression. Cell-specific expression analysis of *Tnf*, *Il-1β*, *Il-6*, and *Ccl2* after 5 days of cuprizone demonstrates that astrocytes are the main source for these cytokines [75]. Together, it is likely that CNS-derived processes alter BBB integrity during cuprizone treatment which may permit the peripheral immune system to exert effects within the CNS.

Another important barrier in the brain exists between the blood and the cerebrospinal fluid, formed by endothelial cells of the choroid plexus (CP). The CP is the major producer of cerebrospinal fluid, and thus an important interface between the blood and cerebrospinal fluid [260]. The CP was shown to be enlarged [261, 262] and a point of T-cell entry into the CNS during neurodegenerative diseases, such as MS or the EAE model [263, 264]. The CP is altered following cuprizone treatment. For example, Fleischer and colleagues found CP enlargement, increased numbers of CP macrophages and T cells in the CP after two weeks of cuprizone treatment [261]. It is still largely unknown however, how the CP or other CNS barrier like the CNS lymphatic or glymphatic system are altered after cuprizone treatment.

##### Evidence neutrophils contribute to cuprizone demyelination

Neutrophils are innate immune cells that responds to inflammatory stimuli to sense and kill pathogens [265]. In the cuprizone model, neutrophils are only present around week 1 in the corpus callosum (Fig. 1B). Liu and colleagues observed significant CNS localization of neutrophils at day 7 using flow cytometry but did not examine whether neutrophils enter into the CNS parenchyma [238]. Neutrophils are thought to promote cuprizone demyelination as knocking out the neutrophil chemokine receptor, CXCR2, in mice prevents demyelination at week 6 of cuprizone treatment. After 4 weeks of cuprizone treatment, nearly all axons are demyelinated in control mice, while in CXCR2-null mice, almost 60% of axons remain myelinated. Loss of CXCR2 does not affect the loss of MBP and 2’,3’-Cyclic-nucleotide 3’-phosphodiesterase (CNPase) expression within the first 2 weeks of cuprizone treatment, but by weeks 3 and 4 MBP and CNPase expression increases in CXCR2^−/−^ mice, suggesting that loss of CXCR2 protects against a delayed myelin protein loss [238]. Transplanting bone marrow from CXCR2^−/−^ mice into irradiated CXCR2^+/+^ mice prevents cuprizone-induced demyelination and oligodendrocyte death suggesting that the source of CXCR2 cells producing demyelination is of peripheral origins [238]. However, CXCR2 is not exclusively a marker for neutrophils as it is also expressed on CD4^+^ T-cells [266] and macrophages [267]. Using flow cytometry, Liu and colleagues find CXCR2 positive cells were Lymphocyte antigen 6 complex locus G6D (Ly6G) positive neutrophils [238]. Depletion of neutrophils using specific GR1 antibodies also reduces the number of apoptotic cells in the corpus callosum by 3 weeks of cuprizone treatment [238], which is a timepoint with sparse neutrophil presence in the CNS. Taken together, CXCR2 plays a role in demyelination, however, it is unclear whether this factor exclusively acts through neutrophils.

##### T-cells presence during cuprizone

T-cells are a predominant effector of the adaptive immune system. T-cells recognize peptide antigens presented by MHC class I or II. T-cells that express CD4 are known as T helper cells while those expressing CD8 are referred to as cytotoxic T-cells [268]. Historically these cells are not linked to demyelination following cuprizone treatment because the loss of the V(D)J recombination activation gene-1 (RAG-1), which is critical for B- and T- lymphocyte generation [269], does not alter demyelination and T-cell accumulation in the corpus callosum [46, 270]. However, these cells still might have executive functions during cuprizone treatment. Recently, Kaddatz and colleagues found that small numbers of cytotoxic CD8 T-cells, and even fewer CD4 T-cells, accumulate within the corpus callosum of cuprizone treated mice up until week 5 [70] (Fig. 1B). The enrichment of CD8^+^ T-cells is in contrast to EAE, where CD4 T-cells predominate [271]. The CNS entry of T-cells after cuprizone treatment may be different than EAE given the lack of perivascular cuffs, which is a sign of lymphocyte infiltration [272]. T-cells in the demyelinated corpus callosum often express markers of activation [70]. Even after disruption of the BBB with pertussis toxin, CD4^+^ and CD8^+^ T-cells are limited in the corpus callosum of cuprizone animals [273], suggesting that these cells are likely attracted to myelin injury specifically. Recent discoveries found severe atrophy of the thymus and spleen and depletion of CD4^+^ and CD8^+^ T-cells due to apoptosis in mice fed with cuprizone [273, 274], which could explain why T-cells are limited in cuprizone lesions.

Peripheral immune cells account only for a small number of all cells that are present at demyelinating sites following cuprizone intoxication and their presence might indicate a role in demyelination that has not been explored yet. Alternatively, T-cells may be responding to demyelination to help restore homeostasis and promote repair.

### Cuprizone induced cell death: a mixed bag

In the previous sections, we have outlined several etiological hypotheses for cuprizone-induced demyelination that might lead to cell death of the oligodendrocytes. Studying what forms of cell death occur in the cuprizone model may help establish the mechanism of its toxicity. Despite decades of research demonstrating that cuprizone induces cell death, the modalities of cell death during cuprizone intoxication are still unclear. One of the first studied forms of cell death is apoptosis, with its name being coined in 1972 by Kerr, Wyllie, and Currie [275], and unsurprisingly it is also the most common form of cell death studied in the cuprizone model. More recently, however, new forms of cell death have been discovered. Of the ten non-apoptotic forms of cell death described by the Nomenclature Committee on Cell Death [276], only Ferroptosis, Necroptosis, and Pyroptosis have been studied after cuprizone ingestion and are reviewed in turn below.

#### Apoptosis

Apoptosis is a programmed cell death induced by both extracellular and intracellular cues. These cues activate caspase 3 by proteolytic cleavage through other caspase isoforms. Once activated, cleaved caspase 3 (CC-3) activates endonucleases and proteases, which ultimately leads to chromatin condensation, reorganization of the cytoskeleton, and cellular disintegration into apoptotic bodies [277].

Oligodendrocytes undergo apoptosis during cuprizone toxicity. Oligodendrocyte apoptosis, based on CC-3 staining, starts as early as day 2 on the cuprizone diet, with extensive depletion of mature oligodendrocytes by day 4 in the corpus callosum [100]. Oligodendrocyte apoptosis reaches its maximum between week 1 and week 3, with the number of apoptotic cells remaining constant thereafter [100, 161, 278]. Hesse and colleagues measured cell death based on nuclear morphology and found that by the end of the first week of cuprizone consumption, dying oligodendrocyte nuclei are almost all condensed, fragmented, and co-label with CC-3 [278]. However, by three weeks of cuprizone consumption, dying oligodendrocytes condense, but are unfragmented, and no longer co-label with CC-3 [278]. The presence of a cell death morphology that lacks CC-3 suggests that apoptosis may shift to a non-CC3 dependent form. Indeed, we found that the condensed nuclear morphology without fragmentation is indicative of a lytic form of cell death [279].

#### Ferroptosis

Ferroptosis is a caspase-independent form of cell death caused by accumulation of toxic lipid peroxidases and free iron. Free iron can react via the Fenton reaction (with H_2_O_2_ from mitochondria), to generate hydroxyl radicals [280] and promote lipid peroxidation [281]. Products of lipid peroxidation, such as MDA and 4-HNE, are highly cytotoxic and serve as common markers of ferroptosis [282, 283].

Iron homeostasis is dysregulated in the liver and brain of mice fed with cuprizone, as several iron metabolism enzymes like hepcidin, transferrin receptor 1 and 2, ferritin heavy chain, and mitochondrial iron transport proteins are dysregulated in the corpus callosum compared to control animals [284, 285]. Dysregulation of these enzymes alters the intracellular iron pool, which can lead to ferroptosis [286]. Cuprizone-mediated reduction in oligodendrocytes between days 1–4 coincides with reduction of the iron storage protein ferritin and an increase of the ferroptosis marker nuclear receptor coactivator 4 (NCOA4). NCOA4 is involved in ferritin breakdown and leads to increased free iron [287]. At the same time, markers of ferroptosis and oxidative stress such as transferrin receptor 1, COX-2, heme oxygenase-1, heat shock protein beta 1, hephaestin, and superoxide dismutase 1 are also dysregulated, suggesting ongoing ferroptosis. However, not all ferroptotic cells are oligodendrocytes as 25% of NCOA4^+^ cells are not oligodendrocytes during cuprizone intoxication [101].

Signs of ferroptotic oligodendrocyte death are present early after cuprizone ingestion. Cuprizone-induced lipid peroxidation due to free radicals begins at 2 days post-cuprizone and remains high throughout the first 4 weeks. Endogenous free radical scavengers are meant to scavenge free radicals, which are crucial for lipid peroxidation. One important ROS scavenger is the glutathione system, which includes two key enzymes: glutathione peroxidase 4 (GPX4) and cystine/glutamate antiporter system x_c_^−^ (Sx_c_^−^). GPX4 reduces toxic lipid peroxides to harmless lipid alcohols, by oxidation of GSH to glutathione disulfide [288–290]. Sx_c_^−^ is a cysteine/glutamate antiporter, which ensures a constant supply of L-cysteine into the cell for GSH synthesis [291]. Cuprizone diet reduces both GPX4 and Sx_c_^−^ and thus reduces the scavenging capacity of lipid peroxides between 2 and 7 days after cuprizone ingestion [101]. Daily injection of ferrostatin—a lipid ROS scavenger [286]—markedly reduces oligodendrocyte death and 4-HNE accumulation at two days and two weeks of cuprizone consumption [101]. Ferrostatin and cuprizone treatment for two weeks also limits myelin loss at four weeks compared to mice treated with only cuprizone for two weeks. The fact that blocking of ferroptosis could prevent long-term myelin loss suggests an important role of ferroptosis in early oligodendrocyte loss in the cuprizone model.

#### Necroptosis

Necroptosis is a regulated form of necrosis triggered by intra- and extracellular signals and is initiated by chemokine receptors such as FAS [292], by pathogen recognition receptors such as Toll-like receptor (TLR)3, TLR4, and Z-DNA binding protein (ZBP)1 [293, 294], and by the TNF receptor (TNFR)1 [295]. Activation of TNFR1 by TNFα leads to the eventual activation of receptor interacting serine/threonine kinase (RIPK)3 via RIPK1. RIPK3 phosphorylates mixed lineage kinase domain-like protein (MLKL) to initiate the oligomerization and translocation of a RIPK3, RIPK1, MLKL complex to the cell membrane where it permeabilizes the cell membrane [296, 297]. Necroptosis is inhibited by caspase-8, a key enzyme for apoptosis, and thus the absence of caspase-8 is required for RIPK3-mediated necroptosis [298]. Given that oligodendrocytes express low levels of caspase-8 [299], they are more susceptible to necroptotic cell death. In the corpus callosum of cuprizone-fed mice, overall RIPK1 levels and RIPK1^+^ oligodendrocytes increase until week 5 of treatment. The administration of 7 N-1, an analog of necrostatin-1 which inhibits RIPK1 [300], reduces the density of RIPK1^+^ oligodendrocytes [301]. 7 N-1 treatment of cuprizone-fed mice also reduces oligodendrocytes loss and increases motor function in animals after 5 weeks. Administration of 7 N-1 three weeks after beginning of cuprizone treatment rescues some of the motor defects, thus linking demyelination and motor outcomes [301]. Similarly, mice lacking RIPK3 display less corpus callosum demyelination compared to WT [301]. However, necroptosis inhibition with RIPA-56, an alternative RIPK1 inhibitor [302], does not prevent cuprizone-induced demyelination but does block the progression of EAE [303]. Inhibition of RIPK1 decreases demyelination only when inhibited by 7 N-1 but not with RIPA-56, which both prevent necroptosis [300, 304], suggesting that RIPK1 might have multiple functions during cuprizone-induced demyelination.

Necroptosis requires the permeability of the cell membrane, which currently, is only indirectly measured by assessing RIPK1/RIPK3/MLKL3 oligomerization. However, these necroptotic proteins may have functions outside of traditional necroptosis. One alternative downstream effect of RIPK1/RIPK3 is the PGAM5-dependent activation of dynamin-related protein 1 (Drp1) [305, 306], a crucial protein for mitochondrial fission [307]. Drp1 hyperactivation induces a variety of cellular damage and is involved in several forms of cell death, including apoptosis [308, 309], necrosis [305, 310], and autophagic cell death [311, 312]. Cuprizone induces DRP1 translocation to the mitochondria, indicative of its hyperactivation, after one week on the diet, which coincides with the peak of oligodendrocyte apoptosis. Treatment of cultured oligodendrocytes with H_2_O_2_ and TNFα induces mitochondrial translocation of Drp-1 and mitochondrial fragmentation [305]. Pharmacological inhibition of Drp-1 in the cuprizone model mitigates MBP and oligodendrocyte loss [306]. Despite the downstream activation of Drp1 by RIPK1/RIPK3/MLKL, necroptosis does not require activation of Drp1 [313–315], suggesting that Drp1-mediated mitochondrial deficiency is a byproduct, and not a direct cause, of necroptosis.

#### Inflammasome mediated cytokine release/pyroptosis

The canonical inflammasome pathway is activated by cytosolic inflammasome sensors such as LRR- and pyrin domain-containing protein (NLRP)3. NLRP3 (reviewed in [316]) detects pathogen associated molecular patterns (PAMPs) or factors released from damaged/dying cells [317–320] and activates caspase-1 (casp-1), the executing caspase in the canonical inflammasome pathway [320]. Activated casp-1 forms a heterodimer complex that catalyzes the maturation of IL-1β and IL-18 [321]. At the same time, casp-1 mediates pyroptotic cell death by cleavage of Gasdermin-D [322]. Cleavage of Gasdermin-D by casp-1 leads to the release of its N-terminal “death domain” [322, 323]. This death domain oligomerizes with components of the cell membrane, forming a lytic pore that results in the release of cellular content [324]. The lytic complex formed by cleaved Gasdermin-D does not necessarily lead to cell lysis but is required to release the cytokines IL-1β and IL-18 [325, 326]. Additionally, Gasdermin-D also cleaves pro-IL-1α to permit the release of mature IL-1α [327]. In the absence of Gasdermin-D, casp-1 activation leads to apoptosis, which can be followed by secondary pyroptosis. In vitro experiments suggest that this secondary pyroptosis is mediated by Gasdermin-E, instead of Gasdermin-D [328]; Brie and colleagues found casp-1 and cleaved Gasdermin-D mediated pyroptosis of oligodendrocytes and microglia in the EAE model and within brain lesions of people with MS [329]. In the cuprizone model, the role of Gasdermin-D has not been explored yet.

In the cuprizone model, NLRP3 expression increases by week 3 and remains high through to week 5 [244]. NLRP3 and NLR Family CARD Domain Containing (NLRC)4, another inflammasome sensor [330], knockout reduces microglia and astrocyte numbers and attenuates demyelination during cuprizone treatment [244, 331, 332]. NLRC4 is expressed highly in astrocytes and microglia and is necessary for the secretion of IL-1β in both cell types after acute demyelination with LPC [332] and may be important for cuprizone-mediated demyelination as well. The activated inflammasome triggers oligodendrocyte death, demyelination, microglia infiltration, and astrocyte activation in the cuprizone model; knockout of *IL-18*, *NLRP3*, *NLRC4*, and *Casp-1* in mice yields less severe demyelination after cuprizone treatment [244, 332]. Saito and colleagues recently described elevation of casp-1 expression levels alongside NLRP3 in the cerebellum of mice fed cuprizone for 6 weeks [333]. Inhibition of casp-1 with VX‐765 reduces the severity of inflammation and behavioral deficits in the EAE model [329]. Casp-1 inhibition with VX‐765 in cuprizone-fed mice significantly reduces the number of Iba-1 positive cells and prevents loss of MBP. Cuprizone-fed mice treated with VX‐765 also have greater levels of spared oligodendrocytes compared to animals not treated with VX-765 [333]. Taken together, inflammasome activation executes critical roles for cuprizone-induced demyelination, though it remains unclear how prominent inflammasome activation results in pyroptotic cells in the cuprizone model.

### Cause of cuprizone-induced demyelination

The cuprizone model is in many ways an ideal system to understand innate immunity in the context of demyelination. Astrocytes and microglia collaborate through the release of cytokines, chemokines, and other, yet to be identified factors, to drive demyelination [79, 107], preventing the release of specific cytokines or chemokines, restricting astrocyte reactivity, or ablating microglia all reduce cuprizone-induced demyelination [107, 181, 189, 243]. Yet many questions remain. For example, what are the contributions of cuprizone itself or its copper chelation in the brain to the ongoing demyelination? Alternatively, are chelation independent mechanisms or cuprizone metabolites the cause for oligodendrocyte death. Does cuprizone act as a toxic factor by altering metabolism and ROS production to trigger oligodendrocyte pathology?

**Fig. 5:**
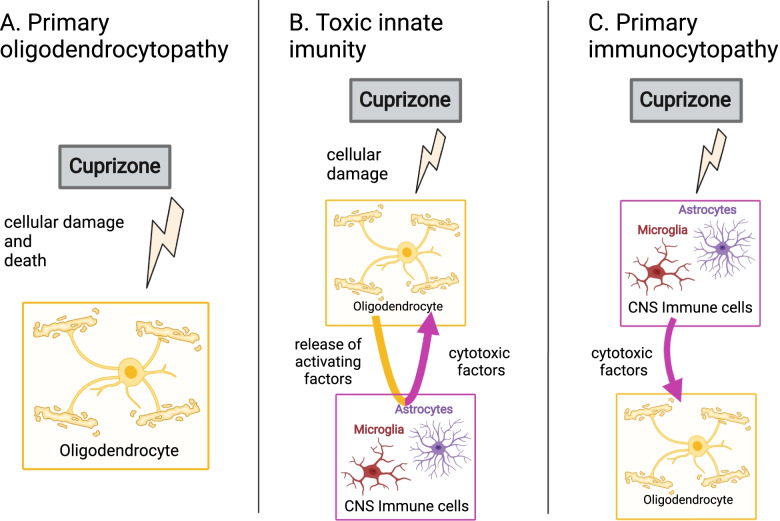
Hypothesis on different pathologies in the cuprizone model: **A** Primary ‘oligodendrocytopathy’, where the effects of cuprizone on the oligodendrocyte population induces cell death. **B** ‘Toxic innate immunity’, where innate immune cells of the CNS take on a cytotoxic phenotype towards the oligodendrocyte population as a reaction to an initial damage to oligodendrocytes. **C** ‘Primary immunocytopathy’, in which the effect of cuprizone on CNS innate immune cells triggers a cytotoxic phenotype of those cells towards oligodendrocytes, without prior damage to the oligodendrocyte population

Early after cuprizone administration, ROS production increases coincident with mitochondrial and metabolic dysfunction that could directly cause oligodendrocyte death [28, 29, 101, 243]. We term this initial trigger of cuprizone-induced oligodendrocyte death as a primary ‘oligodendrocytopathy’ (Fig. 5A). Following this initial oligodendrocyte destruction, an immune response mediated primarily by astrocytes and microglia seems to amplify the demyelination. We call this cascade of destructive, innate inflammation involving microglia and astrocytes ‘toxic innate immunity’ (Fig. 5B). It may also be possible that cuprizone induces an innate immune response where cuprizone intake or the subsequent metabolic processes activate innate immune cells and direct them to induce oligodendrocyte toxicity, which we term ‘primary immunocytopathy’ (Fig. 5C). If and how cuprizone affects astrocytes and microglia directly is still largely unexplored. Importantly, those are not mutually exclusive—on the contrary, we think it highly likely that these mechanisms occur simultaneously.

## Conclusion

Cuprizone intoxication leads to myelin loss and selective oligodendrocyte death in several brain regions with demyelination pathology that displays a striking resemblance to lesions from progressive stages of MS. This demyelination is accompanied by expansion and transition of CNS-derived microglia and astrocytes to become more reactive. Cuprizone intake also results in BBB breakdown and a limited infiltration of peripheral immune cells. Although cuprizone-induced oligodendrocyte loss was historically attributed solely to primary oligodendrocyte death, we now know that CNS inflammation either causes or aggravates the demyelination. We argue that two distinct, yet overlapping and sequential, modes of action within the CNS propagate demyelination. The first mode of action stems from oligodendrocyte intrinsic mechanisms, which we have termed a ‘primary oligodendrogliapathy’, wherein cuprizone alters mitochondrial metabolism and the synthesis of myelin components which eventually leads to cell death. The second mode of action stems from oligodendrocyte extrinsic inflammatory mechanisms within the CNS by both resident and peripherally-derived immune cells. These cells adopt a pro-inflammatory phenotype, either as a direct response to cuprizone, which we have termed a ‘primary inmmunocytopathy’, or by molecules released from dying oligodendrocytes, refered as ‘toxic innate immunity’. In the latter case, reactive astrocytes produce cytokines and chemokines that are directly cytotoxic to oligodendrocytes or attract and stimulate microglia. Reactive microglia are vital for phagocytosis of myelin debris, which inhibits remyelination, but are also necessary for demyelination as their ablation prevents demyelination after cuprizone intoxication. Although oligodendrocytes are primarily the victims of demyelination, they might themselves contribute to the proinflammatory environment by release/secretion of cytokines and chemokines and/or the expression of MHC-II molecules for antigen presentation and further amplified immune cell activation. Other parts of the innate immune response, such as the complement system may have direct cytotoxic effects on oligodendrocytes and attract microglia and astrocytes. Peripheral immune cells such as T-cells are present at the site of demyelination, but they are likely not promoting demyelination.

Cuprizone intake causes the loss of oligodendrocytes by a variety of different cell death forms. Apoptosis is the most commonly studied type of cell death in the cuprizone model as it is well understood and easy to assess. However, we know now that other forms of cell death, such as necroptosis, ferroptosis, and inflammasome mediated cell death/pyroptosis lead to oligodendrocyte death in the cuprizone model and occur at different time points after intoxication. For example, apoptosis and ferroptosis occur up until week 2 – 3 of cuprizone intoxication while other types of cell death seem to contribute to oligodendrocyte loss starting at week 2 – 3, concomitant with myelin loss and accumulation of astrocytes and microglia. A shift from apoptotic/ferroptotic cell death to other forms might also indicate a shift in the underlying mechanisms of cell death. To better understand the complexity of the cuprizone model, it is critical to explore the earliest time points of cuprizone treatment, particularly when microglia and astrocyte activities are impaired. We believe that resolving the time-dependent pathways that underlie the various types of cell death will be vital to better understanding the mechanisms of cuprizone toxicity.

As early loss of oligodendrocytes appears sufficient to drive late-stage myelin loss [101], it is critical to understand what factors drive oligodendrocyte loss in the first week of cuprizone treatment. Although we continue to better understand the mechanisms of cuprizone toxicity, there are still many unresolved questions surrounding this model. Is astrocyte and microglia reactivity secondary to an initial oligodendrocyte insult or does cuprizone per se induce a toxic pro-inflammatory phenotype in those cells? How much do oligodendrocytes themselves contribute to their downfall? Also, regarding the interaction of cuprizone with cellular components many questions remain. How widespread is the copper-chelation independent protein inhibition and could this be a reason for oligodendrocyte death? To date, it is unclear if cuprizone is metabolized in the body and if a potential metabolites cause oligodendrocyte death. By rigorously identifying how cuprizone induces demyelination, we may find new parallels with human white matter diseases, such as MS, and be able to use this model more efficiently in preclinical studies.

## Data Availability

Not applicable.

## Abbreviations

4-HNE: 4-Hydroxynonenal
AIF-1: Apoptosis-inducing factor-1
APAF-1: Apoptotic protease activating factor-1
ATP7: Copper-transporting P-type ATPase
BBB: Blood–brain-barrier
Casp-1: Caspase-1
CC-3: Cleaved caspase 3
CCL: C–C motif chemokine ligand
CCR: C–C motif chemokine receptor
CD: Cluster of differentiation
CGT: Ceramide galactosyltransferase
CNS: Central nervous system
CNPase: 2’,3’-Cyclic-nucleotide 3’-phosphodiesterase
COX: Cyclooxygenase
CP: Choroid plexus
Crry: Complement receptor 1-related Gene/Protein y
CSF: Colony stimulating factor
CSF1R: Colony stimulating factor 1 receptor
CTR1: High affinity copper uptake protein 1
CXCL: C-X-C motif chemokine ligand
CXCR: C-X-C motif chemokine receptor
Cyt c: Cytochrome c
DNA: Deoxyribonucleic acid
Drp1: Dynamin-related protein 1
EAE: Experimental autoimmune encephalomyelitis
GFAP: Glial fibrillary acidic protein
GLAST: Glutamate aspartate transporter
GPX4: Glutathione peroxidase 4
GSH: Glutathione
HMG-CoA: β-Hydroxy β-methylglutaryl-CoA
IKK2: Inhibitor kappa B kinase 2
IL: Interleukin
INF: Interferon
IRF-8: Interferon regulatory factor 8
LIF: Leukemia inhibitory factor
LPC: Lysophosphatidylcholine
LPS: Lipopolysaccharide
Ltα: Lymphotoxin α
MAG: Myelin-associated glycoprotein
MBP: Myelin basic protein
MDA: Malondialdehyde
MERTK: MER proto-oncogene, tyrosine kinase
MHC: Major histocompatibility complex
MLKL: Mixed lineage kinase domain-like protein
MRI: Magnetic resonance imaging
mRNA: Messenger ribonucleic acid
MS: Multiple Sclerosis
NCOA4: Nuclear receptor coactivator 4
NF-κB: Nuclear factor kappa B
NLRC: NLR Family CARD domain containing
NLRP: LRR- and pyrin domain-containing protein
NMDAR: N-Methyl-D-aspartic acid receptors
OPC: Oligodendrocyte progenitor cell
OSM: Oncostatin M
P75NTR: P75 neurotrophin receptor
PAMPs: Pathogen associated molecular patterns
PARP: Poly (ADP-ribose) polymerase
PDGF: Platelet-derived growth factor
PG: Prostaglandins
PGE2-EP2: PG E2 receptor 2 subtype
PPMS: Primary-progressive MS
PrPC: Cellular prion protein
RAG-1: Recombination activation gene-1
RIPK: Receptor interacting serine/threonine kinase
ROS: Reactive oxygen species
RRMS: Relapsing–remitting MS
sCrry: Soluble Crry
SPMS: Secondary-progressive MS
Sxc-: Cystine/glutamate antiporter system xc −
TLR: Toll-like receptor
TMEV: Theiler’s murine encephalomyelitis virus
TNF: Tumor necrosis factor
TNFR: TNF receptor
TREM: Triggering receptor expressed on myeloid cells
TrkB: Tropomyosin receptor kinase B
VgluT: Vesicular glutamate transporter
ZBP: Z-DNA binding protein
